# Effects and safety of *Psilocybe cubensis* and *Panaeolus cyanescens* magic mushroom extracts on endothelin-1-induced hypertrophy and cell injury in cardiomyocytes

**DOI:** 10.1038/s41598-020-79328-5

**Published:** 2020-12-18

**Authors:** Sanah M. Nkadimeng, Christiaan M. L. Steinmann, Jacobus N. Eloff

**Affiliations:** 1 Phytomedicine Programme, Paraclinical Sciences Department, University of Pretoria, P/Bag X04, Onderstepoort, Pretoria, 0110 Gauteng South Africa; 2 Physiology Department, School of Medicine, Sefako Makgatho Health Sciences University, Pretoria, South Africa

**Keywords:** Cell biology, Medical research

## Abstract

Prevalence of major depression in people with chronic heart failure is higher than in normal populations. Depression in heart failure has become a major issue. Psilocybin-containing mushrooms commonly known as magic mushrooms, have been used since ancient times for their mind healing properties. Their safety in cardiovascular disease conditions is not fully known and may pose as a risk for users suffering from these illnesses. Study investigates the effects and safety of *Psilocybe cubensis* and *Panaeolus cyanescens* magic mushrooms use from genus *Psilocybe* and *Panaeolus* respectively, in a pathological hypertrophy conditions in which endothelin-1 disorder is a contributor to pathogenesis. We examined the effects of the mushrooms extracts on endothelin-1-induced hypertrophy and tumor necrosis factor-α (TNF- α)-induced cell injury in H9C2 cardiomyocytes. Mushrooms were oven dried and extracted with cold and boiling-hot water. H9C2 cardiomyocytes were induced with endothelin-1 prior to treatment with extracts over 48 h. Cell injury was stimulated with TNF-α. Results proposed that the water extracts of *Panaeolus cyanescens* and *Psilocybe cubensis* did not aggravate the pathological hypertrophy induced by endothelin-1 and also protected against the TNF-α-induced injury and cell death in concentrations used. Results support medicinal safe use of mushrooms under controlled conditions and cautioned use of higher concentrations.

## Introduction

Heart failure is well-defined as a complex clinical syndrome that may results from any structural and/or functional cardiac disorder that impairs the ability of the heart ventricle to fill with or eject blood as needed^1^. Heart failure is a public health problem and a leading cause of morbidity and mortality^1^. The disease imposes a significant impact on the quality of life leading to disruptions in daily life on many affected persons^2^. It is reported that prevalence of major depression in chronic heart failure is about 20–40%, which is 4–5% higher than in general populations^3^. Furthermore, depression in heart failure patients may lead to a two-fold increase in mortality^3^. As a result, depression in patients with heart failure has become a major problem^3^.

Psilocybin-containing mushrooms commonly known as magic mushrooms have been used since ancient times for their mind healing properties in different indigenous populations^4–6^. Recently, psilocybin (4-phosphoryloxy-N,N-dimethyltryptamine), a natural hallucinogen and a main compound in magic mushrooms was found to have significant antidepressant effects^7^. Awareness and use of magic mushrooms for depression and improved quality of life is growing as a result. The recreational dose by most users ranges from 1 to 3.5 g of dried mushrooms or 10–15 g of fresh mushrooms^8^. Magic mushrooms are generally not considered toxic with lethal dose of 280 mg/kg in rats and 17 kg/70 kg for humans^8^. Fatal intoxications due to exposure to magic mushrooms are rare and often reported to be mainly due to combination with other drugs^8^. However, psilocybin-containing mushrooms are also known to induce temporary increase in heart rate and blood pressure^7,8^. This increase in heart rate and blood pressure may present risk to users suffering from cardiovascular illnesses such as heart failure. Therefore use and safety of magic mushrooms in heart failure conditions needs to be investigated.

The study investigates the effects and safety of *Psilocybe cubensis* and *Panaeolus cyanescens* magic mushrooms from genus *Psilocybe* and *Panaeolus* respectively, in pathological hypertrophy conditions in which endothelin-1 (ET1-1) disorder is a contributor to pathogenesis. Endothelin-1 is a potent vasoconstrictor that plays a critically important role in the induction of myocyte hypertrophy^9^. Cardiac hypertrophy involves an increase in heart size without myocytes proliferation^10^. Initially cardiac hypertrophy is adaptive but overtime as the disease continues it moves into a decompensation stage which is pathological and progresses into heart failure^11^. Pathological hypertrophy is characterised by increase in expressions of foetal genes such as natriuretic peptides (ANP) and brain natriuretic peptides (BNP) consequently, both ANP and BNP are well-known hall marks of heart failure^9^. Involvement of G_q_-protein couple receptor signalling is another main indicator of pathological hypertrophy^12^. Their agonists include ET-1 and angiotensin II both of which are chronically increased in the illness where they induce their effects on cardiac myocytes, altering excitation contraction coupling and more chronic effects on cardiac growth^13,14^. Potent pro-hypertrophic effects of ET-1 in vitro on primary cultures of ventricular myocytes are noticeably within the first 15 min of its application on the cells^9^.

This study evaluates for the first time the effects of *Psilocybe cubensis* and *Panaeolus cyanescens* magic mushrooms water extracts, one of the commonly used extraction method by mushroom users, on ET-1-induced hypertrophy using the rat embryonic ventricular H9C2 cardiomyoblast, which is a well-known and extensively used in vitro cell model with widely accepted reliability in cardiovascular drug discovery^15^. We also evaluate the safety of the two magic mushrooms on tumor necrosis factor-α (TNF-α)-induced cell injury and death on the H9C2 cardiomyocytes. Tumor necrosis factor-α is a pro-inflammatory cytokine also known to plays a crucial role in the pathogenesis and progression of cardiovascular injury and apoptosis, hypertrophy and heart failure^16^. The result from this study may provide information on the safety or risk of the two magic mushroom uses in major depression associated with heart failure conditions.

## Results

### Effects on cell width measurements and BNP concentrations

**Figure 1:**
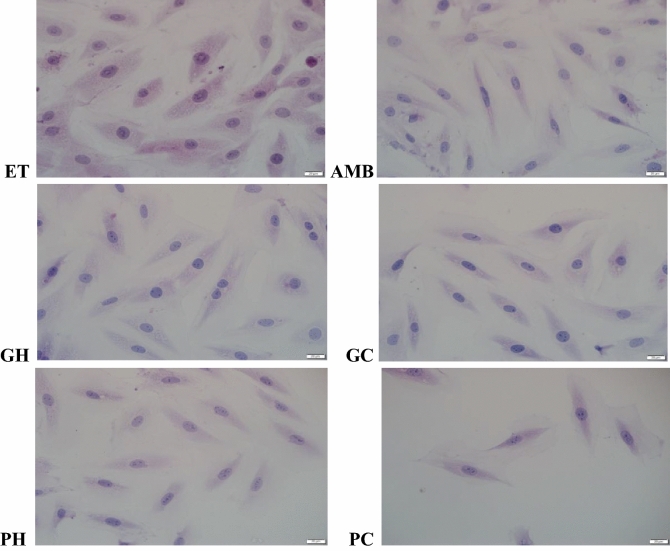
Effects of the hot-water (GH) and cold-water (GC) extracts of *P. cubensis* and hot-water (PH) and cold-water (PC) extracts of *Pan cyanescens* mushrooms (50 μg/mL) and positive control ambrisentan (AMB) (25 μg/mL) on the morphology of the cells after 48 h treatment, repeated in three different times.

**Figure 2:**
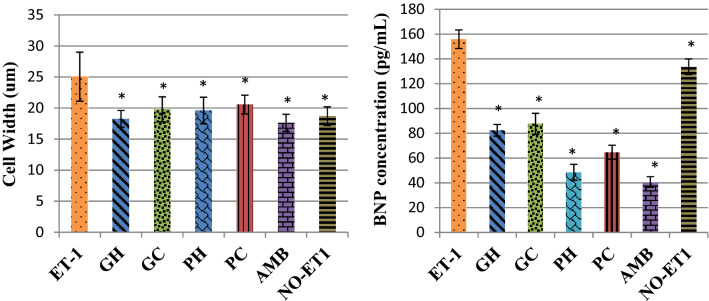
Effects of the hot-water (GH) and cold-water (GC) extracts of *P. cubensis* and hot-water (PH) and cold-water (PC) extracts of *Pan cyanescens* mushrooms (50 μg/mL) and positive control ambrisentan (AMB) (25 μg/mL) on cell width size measurements and BNP levels after 48 h treatment, repeated in three different times [*significant].

Morphological analysis showed that endothelin-1 stimulation increased the cell sizes of the cells and the positive control ambrisentan reversed the cell sizes, Fig. 1. The hot-water and cold-water of the two mushrooms, *P. cubensis* and *Pan cyanescens* reduced the sizes of the cells similar to ambrisentan. The control cells that were induced with ET-1 increased significantly (*p* < 0.0001) in cell width measurements when compared to the non-induced NO-ET1 cells, Fig. 2. The hot-water (GH) (*p* < 0.0001) and cold-water (GC) (*p* < 0.0001) extracts of *P. cubensis* and *Pan cyanescens* hot-water (PH) (*p* < 0.0001) and cold-water (PC) (*p* < 0.0001) extracts significantly reduced the cell with sizes of the treated cells when compared with ET-1 control cells. The positive control ambrisentan also significantly (*p* < 0.0001) reduced the cell sizes in comparison to the ET-1 cells.

The control ET-1 induced cells significantly (*p* < 0.010) increased the BNP concentrations in comparison to the NO-ET1 non-induced cells, Fig. 2. The positive control ambrisentan reduced the ET-1 effect significantly (*p* < 0.0001) compared to the ET-1 control. The *P. cubensis* hot-water (GH) and cold-water (GC) extracts significantly decreased the BNP (*p* < 0.0001 and *p* < 0.0001 respectively) in comparison to the ET-1 control. *Pan cyanescens* hot-water (PH) and cold-water (PC) extracts also significantly decreased the levels of BNP with *p* < 0.0001 and *p* < 0.0001 respectively when compared to the control ET1 cells, Fig. 2.

### Effects on mitochondrial activity

**Figure 3:**
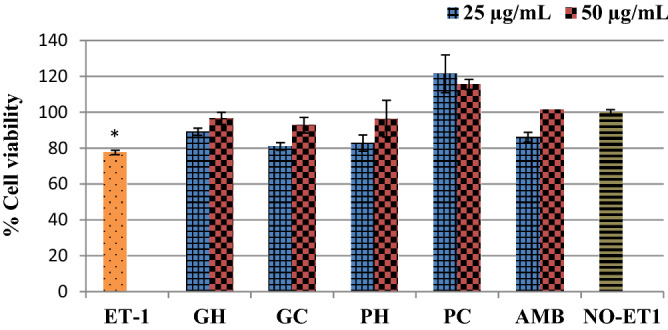
Effects of the hot-water (GH) and cold-water (GC) extracts of *P. cubensis* and hot-water (PH) and cold-water (PC) extracts of *Pan cyanescens* mushrooms (25 and 50 μg/mL) and positive control (AMB) ambrisentan (10 and 25 μg/mL) on % cell viability after 48 h treatment. Repeated in three different times [*significant].

Treatment with ET-1 reduced mitochondrial activity of the cells significantly (*p* < 0.0001 indicated by % cell viability below 80% in comparison to the NO-ET1 cells, Fig. 3. The water extracts of *P. cubensis* and *Pan cyanescens* increased the viability of cells above 80% in safe margins in a dose dependant manner same as ambrisentan, positive control, Fig. 3.

### Effects on TNFα- levels

**Figure 4:**
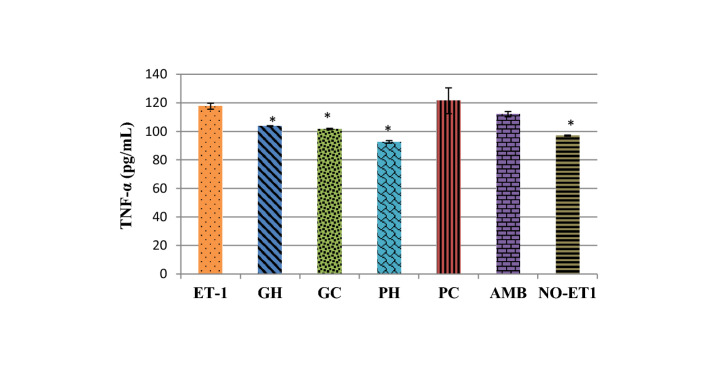
Effects of the hot-water (GH) and cold-water (GC) extracts of *P. cubensis* and hot-water (PH) and cold-water (PC) extracts of *Pan cyanescens* mushrooms (50 μg/mL) and positive control (AMB) ambrisentan (25 μg/mL) on levels of TNF-α after 48 h treatment repeated in three different times [*significant].

The ET-1 stimulation increased significantly (*p* = 0.006) the TNF-α level of the cells compared to NO-ET1 cells, Fig. 4. In comparison to the ET-1 control cells, the hot-water (GH) and cold-water (GC) extracts of *P. cubensis* reduced significantly the TNF-α levels (*p* = 0.047 and *p* = 0.024 respectively). The hot-water (PH) extract of *Pan cyanescens* also significantly (*p* = 0.002) decreased the levels of TNF-α concentrations while the cold-water (PC) increased the levels non-significantly when compared with ET-1 control, Fig. 4 The positive control ambrisentan also reduced the ET-1 induced TNF-α concentration non-significantly.

### Effects on ROS levels and rate of cell growth

**Figure 5:**
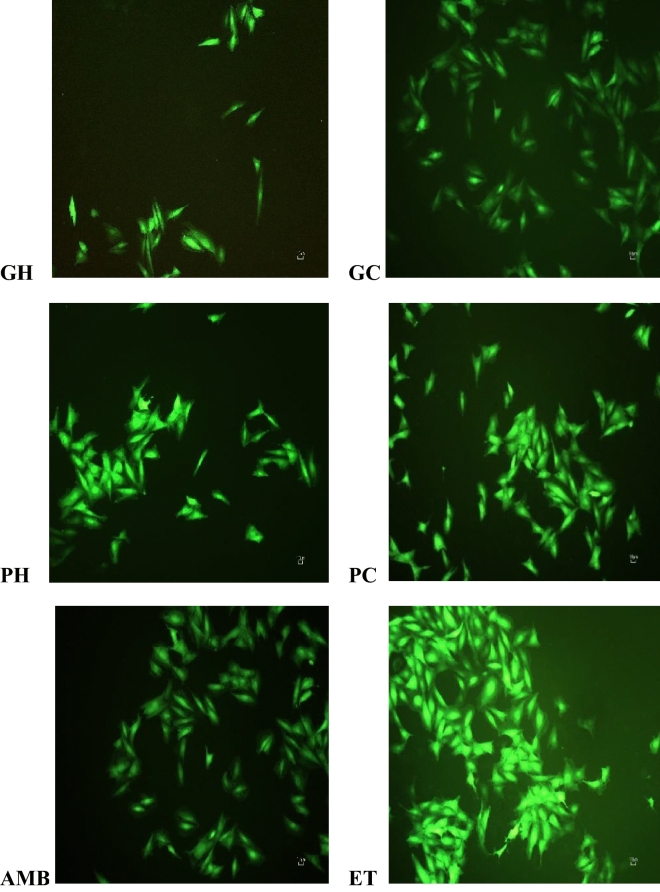
Fluorescence effects of the hot-water (GH) and cold-water (GC) extracts of *P. cubensis* and hot-water (PH) and cold-water (PC) extracts of *Pan cyanescens* mushrooms (50 μg/mL) and positive control (AMB) ambrisentan (25 μg/mL) on intracellular ROS production after 1-h treatment (experiments were done in three different times).

ET-1 increased the intracellular ROS productions (mainly the superoxide and hydroxyl radicals) which were reduced by the positive control ambrisentan, Fig. 5. The water extracts of *Pan cyanescens* reduced the ROS signalling while the water extracts of *P. cubensis* reduced the ROS signal very close to the positive control, Fig. 5.

**Figure 6:**
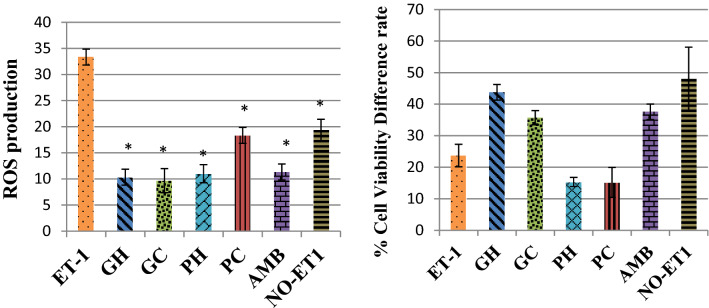
Effects of the hot-water (GH) and cold-water (GC) extracts of *P. cubensis* and hot-water (PH) and cold-water (PC) extracts of *Pan cyanescens* mushrooms (50 μg/mL) and positive control (AMB) ambrisentan (25 μg/mL) on intracellular ROS production measured and % cell viability growth rate after 1-h treatment, repeated in three different times [*significant].

Treatment with ET-1 increased intracellular ROS significantly (*p* < 0.0001) in comparison to the NO-ET1 non-induced cells, Fig. 6. The positive control ambrisentan significantly decreased (*p* < 0.0001) the ET-1 induced ROS effect. *P. cubensis* hot-water (GH) and cold-water (GC) extracts significantly decreased the ROS production (*p* < 0.0001 and *p* < 0.0001 respectively) in comparison to the ET-1 control. *Pan cyanescens* hot-water (PH) and cold-water (PC) extracts also significantly decreased the levels of ROS production with *p* < 0.0001 and *p* < 0.0001 respectively when compared to the control ET1 induced cells, Fig. 6.

The effect of ET-1 stimulation and extract treatment on the rate of cell growth as indicated by % cell viability was also determined for each sample, Fig. 6. The extracts and ET-1 increased growth of cells after stimulation and treatment when compared to cell viability before exposure. The results also showed that the ET-1 stimulated cells lowered rate of growth when compared to NO-ET1 non-stimulated cells. The cold-water (GC) extracts of *P. cubensis* increased rate of cell growth same as positive control ambrisentan and the hot-water (GH) had the highest growth rate when compared to normal NO-ET1 cells, Fig. 6. The two water extracts of *Pan cyanescens* on the other hand induced lower rate of cell growth when compared to NO-ET1 cells. The rate of growth was even lower than ET-1 stimulated control cells, Fig. 6.

**Figure 7:**
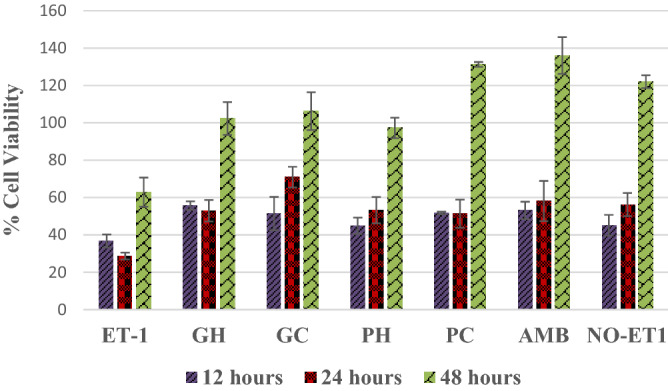
Effects of the hot-water (GH) and cold-water (GC) extracts of *P. cubensis* and hot-water (PH) and cold-water (PC) extracts of *Pan cyanescens* mushrooms (50 μg/mL) and positive control (AMB) ambrisentan (25 μg/mL) on % cell viability growth rate after 1-h treatment and measured over 12, 24 and 48 h, repeated in three different times.

The effects of the extracts on the growth rate of the cells after 12 h showed an increase in the treatments and positive control compared to the ET-1 treatment, Fig. 7. The % viability growth rate continued to improve in 24 h and such that the cold-water extracts of *Pan cyanescens* mushrooms was the highest of the extracts close to ambrisentan in the 48th hour.

### Effects on TNF-α-induced cell injury and death

**Figure 8:**
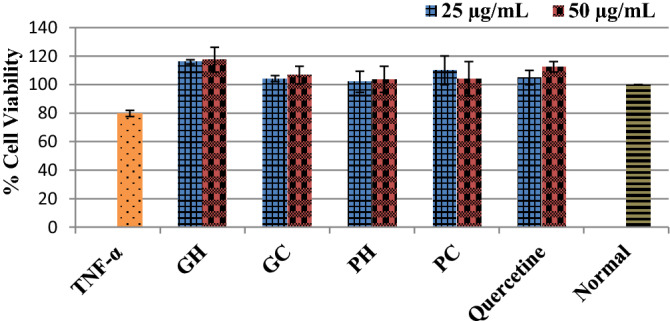
Protective effects of the hot-water (GH) and cold-water (GC) extracts of *P. cubensis* and hot-water (PH) and cold-water (PC) extracts of *Pan cyanescens* mushrooms (25 and 50 µg/mL) and the positive control quercetin (12.5 and 25 µg/mL) on TNF-α-induced cell injury and cardiomyocytes death over 24 h, repeated in three different times [*significant].

Stimulation with TNF- α induced a significant (*p* < 0.0001) decreased % viability of cells below 80% when compared with the normal un-induced and non-treated cells; Fig. 8. The cold and hot-water extracts of *P. cubensis* and *Pan cyanescens* mushrooms increased % cell viability of treated cells above 100% same as the positive control quercetin.

## Discussion

Heart failure is a public health problem that significantly impacts daily management and the quality of life of many affected persons^2^. Major depression in chronic heart failure and its increasing role in heart failure mortality is an additional problem^3^. Although magic mushrooms have been used in ancient and recent times for mind healing and are known to improve the quality of life, their safety in cardiovascular diseases such as heart failure is not known. Our study investigated for the first time, the effects of the hot-water and cold-water extracts of *Panaeolus cyanescens* and *Psilocybe cubensis* magic mushrooms on ET-1, a major physiological inducer of hypertrophic changes in vitro on rat H9C2 cardiomyocytes where we evaluate the safety or ability of the extracts to exacerbate these effects. The in vitro H9C2 cardiomyoblast cells protocol model used in the study was chosen based on their established and proven capacity to exhibit physiological responses useful in drug discovery for cardiovascular medicine^17^. The results from the study demonstrated that treatment with ET-1 increased significantly the cell measurements sizes, BNP levels of the stimulated cells and decreased mitochondrial activity significantly as indicated by cell viability when compared to the non-induced NO-ET1 cells. These effects were in agreement with previous studies indicating successful cellular ET-1-induced hypertrophy in our study^18^.

The water extracts of *P. cubensis* and *Pan cyanescens* mushrooms reduced significantly the ET-1 induced cell size measurements of treated cells same as the positive control, ambrisentan, which selectively blocks ETA receptor and inhibit ET-1 pro-hypertrophic properties. The four water extracts also significantly reduced the ET-1-induced concentrations of BNP, one of the well-known hall mark peptide of heart failure. As a result the four extracts reversed the two main indices of hypertrophy (cell size and BNP levels) induced by ET-1 significantly in the concentrations used. Moreover, the four water extracts of the two magic mushrooms also improved mitochondrial activity of the cells signified by increasing the % cell viability of ET-1-induced cells in a dose-dependent manner same as positive control, ambrisentan indicating safety at the concentrations investigated in the study. Furthermore the two extracts of *P. cubensis* and hot-water extract of *Pan cyanescens* mushrooms reduced the TNF-α concentration in the treated cells compared to ET-1-induced control cells while the cold-water of *Pan cyanescens* slightly increased it non-significantly. TNF-α is a key pro-inflammatory cytokine that is known to promote cardiac dysfunctions and contributes to heart failure^19^. By reducing TNF-α, the three extracts demonstrated potential safety in heart failure conditions.

Many studies have established that ROS plays a very important role in the progression of cardiovascular diseases such as heart failure by inducing oxidative stress which in turn leads to cell and tissue injury^20^. Superoxide and hydroxyl radicals are among the most prominent ROS causing toxic insults to the human body^21^. In our study we measured ROS levels especially superoxide and hydroxyl radicals over 1 h of treatment after 2 h ET-1 stimulation and the results showed that the four water mushroom extracts reversed the ET-1-induced ROS levels significantly same as the positive control when compared to ET-1-induced control cells. By decreasing ROS levels, the extracts demonstrated safety and protective effect against ET-1 induced oxidative stress that will be beneficial in heart failure.

Furthermore, the decrease in ROS observed with the extracts was not due to toxicity based on the positive increase in cell growth rate (Fig. 4) where % viability of cells continued to increase after 1 h treatment. However, it was also quite interesting to perceive the differences between the water extracts of the two mushrooms on cell growth rate analysis in comparison to the NO-ET-1 cells. The cells treated with *P. cubensis* extract after 1 h continued to grow at the rate close to positive control, ambrisentan and NO-ET1 cells while the water extract of *Pan cyanescens* lead to a reduction in rate of cell growth even slower than that observed with ET-1-induced control cells. This effect showed that although *Pan cyanescens* water extracts reduced ROS levels, they also contain other compounds that lowered rate of cell growth. However, it is known that *Pan cyanescens* mushrooms are unique in that they possess very high levels of urea in addition to psilocybin, psilocin, baeocystin, and other compounds generally known to be present in magic mushrooms^22^. Urea is also known to induce cell cycle delay and promote a slow rate of increase of cells in log phase of growth^23^. This could be the reason behind such a reduction in rate of cell growth observed with *Pan cyanescens* water extracts treatment compared to the other samples. However, we also observed an improvement change from 12 h in the growth rate of the two water extracts of *Pan cyanescence* such that the cold water exhibited the highest rate of growth by the 48th hour treatment. Caution is however needed with higher concentrations of *Pan cyanescens* mushroom water extracts as they may have potential to induce cell cycle delay in the first hour after consumption.

To further investigate safety of the extracts on cell injury, the results showed that the cardiomyocytes induced with TNF-α stimulated a significant cell death indicated by reduction in the viability of induced cells below 80% compared to normal non-induced cells. The four extracts of the two magic mushrooms reversed the TNF-α-induced cell injury and death signified by increasing % cell viability of the treated cells same as positive control quercetin in a dose dependent manner. This effect demonstrated the protective effect of the mushroom extracts against cardiomyocyte injury that will be beneficial in a pathological hypertrophy condition.

Moreover, it was also interesting to observe that although the cold-water extract of *Pan cyanescens* mushroom did not inhibit production of TNF-α concentration in the ET-1 stimulated cells after 48 h, the extract still protected against ET-1 induced cell death by increasing % cell viability of cells even higher than the positive control and non-induced cells at the concentrations used in the study as shown in Fig. 3. This effect combine with the protective effect of the cold-water extract on TNF-α-induced cell injury above demonstrate that the extract may have compounds that blocked the induced-cardiomyocyte apoptosis cascades probably by activating or promoting expressions of the cell death repressors. Studies have shown that the apoptosis effects of TNF-α in the heart is depending on the type of its receptor whereby it exhibits its cardiotoxic effect through its receptor TNFR1 (tumor necrosis factor receptor1)^24,25^. After binding to its TNFR1 receptor, TNF-α can stimulates apoptosis in cardiomyocytes by activating sphingomyelin signal transduction pathway leading to production of the intracellular signalling molecule, sphingosine^25^.

Sphingosine is a well-known effective inducer of apoptosis on cardiomyocytes and it induces its effect by down-regulating the expressions of cell death repressors, Bcl-2 (B Cell Lymphoma-2) family protein in the same manner as it does in other cell types^25^. Furthermore, sphingosine is also a potent inhibitor of protein kinase C (PKC), which has been found to protect cells from apoptotic cell death; consequently, sphingosine may promote apoptosis through PKC inhibition by changing the level of Blc-2 phosphorylation^25^. Moreover, many studies have also found that the beta-adrenergic receptor1 blocker (β_1_-blocker) enhances the resistance of cardiomyocytes to cell death by expanding the survival range of the switching response of Bcl-2^26^. Beta-adrenergic receptor1 is one of the β-adrenergic receptors known to transduce the cell death signal via cyclic 3′,5′-adenosine monophosphate (cAMP)-dependent signalling pathways of cardiomyocytes which may result in the reduction of cardiac contractility related to the pathophysiology of heart failure^26^. We propose possibility that the water extracts of *Pan cyanescens* and *P. cubensis* mushrooms may possess compounds with potential ability to promote or activate overexpression and/or phosphorylation of Blc-2 proteins pathways thereby inhibiting the induced-apoptosis and preserving mitochondrial membrane integrity of the treated cells. And this compound/s may be more pronounced in the cold-water extraction of *Pan cyanescens* mushroom.

Moreover, the suppressive effects of the two-water extract of *P. cubensis* and the hot-water extract of *Pan cyanescens* on ET-induced TNF-α levels of treated cells also indicated that these extracts may also have activity on the nuclear factor (NF)-κB signalling, a transcription factor that regulates the expression of many pro-inflammatory cytokines including TNF-α and the genes associated with apoptosis^27^. Studies have proposed that the inflammation-related NF-κB signaling and its correlation with apoptosis is the underlying mechanism in the pathogenesis of heart failure^28^. Furthermore, oxidative stress may also activate NF-κB and initiate the transcription of numerous pro-apoptotic genes, which includes *Bax*, *Fas* and *Fas ligand*, inducing myocardial cell apoptosis and further promoting heart failure condition^29^. A further study into the mechanisms of action in vitro and in vivo is therefore recommended. Furthermore, in previous studies, mycochemical compounds were verified to be present in both *Pan cyanescens* and *P. cubensis* mushrooms such as alkaloids, with known biological activities including toxicity against cells of foreign organisms^22,30^. Saponins which are known as potent antioxidant that neutralises free radicals and flavonoids with antioxidant, anti-inflammatory and anti-carcinorgenic activities^22,30^. Finally, tannins with antioxidant properties related to their scavenging activities reported to have been used against heart diseases were also detected in the two mushrooms^22,30^. Presence of these compounds could have also played a role in the protective activities exhibited by the water extracts of *Pan cyanescens* and *P. cubensis* mushrooms in the study. The study also showed that in general the cardioprotective effects were more pronounced with the hot-water extracts of the two mushrooms compared to the cold-water extractions suggesting more benefit with users of the mushrooms that consume the mushrooms with tea.

In conclusion, the study demonstrated that ET-1 significantly increased cell size measurements, BNP, TNF-α and ROS levels and decreased mitochondrial activity of the stimulated cardiomyocyte cells. The results indicated that the water extracts of *P. cubensis* and *Pan cyanescens* mushrooms significantly reversed the cell size and BNP levels which are two indices of hypertrophy and increased viability of cells. The two water extracts of *P. cubensis* and hot-water extract of *Pan cyanescens* mushrooms also significantly reduced the ET-1-induced TNF-α, a pro-inflammatory cytokine involved in the progression of pathological hypertrophy and heart failure. The four extracts also inhibited the ET-1 induced intracellular ROS levels significantly indicating potential safety in these conditions. Furthermore, the extracts exhibited protective properties against TNF-α-induced cell injury and death in the concentrations investigated in the study.

Finally, the study proposed that the water extracts of *Panaeolus cyanescens and Psilocybe cubensis* mushrooms did not increase the ET-1-induced hypertrophic changes, instead the two mushrooms had cardioprotective potential properties and also alleviated against TNF-α-induced cell injury and death in the concentrations investigated. The study indicated for the first time the safety and potential beneficial properties of *Panaeolus cyanescens* and *Psilocybe cubensis* mushrooms usage in heart failure conditions where ET-1 is the course of pathological hypertrophic changes. However, cautioned with higher concentrations. Further investigation is required to establish the underlying mechanisms of action.

## Materials and methods

### Ethical and protocol clearances

The protocol for this study was submitted to the University of Pretoria research committee (UPREC) and approved with the number REC045-18. The protocol was also submitted and approved by the Medical Control Council (MCC) committee of South African Health Department and a permit (POS 223/2019/2020) as psilocybin-containing mushrooms are schedule 7 substances in South Africa.

### Growing mushrooms and making extracts

The spores print syringes of *Psilocybe cubensis* (*P. cubensis*) and *Panaeolus (Coplandia) cyanescens* (*Pan cyanescens*) mushrooms commonly known as “Golden teacher” and "Natal blue minie” verified with an SKU number TEA-1 and TBMN-1 respectively by the Sporespot Company and were all purchased from Sporespot Company Durban, South Africa. The sterile substrate with SKU number SSK-2 used to grow the mushrooms was also purchased from the same company. As soon as they arrived, the spores were inoculated in the substrate and allowed to grow in a sterilised monotub with monitored temperature and humidity under sterile conditions. Mushrooms were grown, harvested and extracts with hot boiling and cold water solvent according to Nkadimeng et al.^31^ method. The extracts were kept in dark in the fridge until use. The hot-water and cold-water extracts of *P. cubensis* are referred to as GH and GC respectively while the hot-water and cold-water of *Pan cyanescence* are referred to as PH and PC in the study.

### Culturing of cells

The rat H9C2 cardiomyoblast cells were purchased from American Type Culture Collection and maintained using Dolbecco Modified Eargle media (DMEM) (Pan, Separations Scientific) supplemented with 10% fetal bovine serum (FBS) (Gibco, Sigma Aldrich) and 1% of 100 IUnits/mL penicillin and 100 µg/L streptomycin (Pan, Celtics diagnostic) in 75 cm^2^ tissue culture treated flasks (NEST, Whitehead Scientific). The cells were grown in an incubator (HERAcell 150, Thermo Electron Corporations, USA) at 37 °C in 5% CO_2_ balanced air.

#### Treatment of cells

The H9C2 cardiomyoblast cells were cultured according to the method of^18,32^ with modification. Briefly as soon as cells reached 70% confluency they were passaged, counted and 1 × 10^6^ cells seeded and grown on glass cover slips in 6 well plates (NEST, Whitehead scientific). After 24 h medium was removed, the cells in the 6 well plates were deprived of serum for 18 h. After 18 h sera-free media was removed, and the cells were treated with 100 nM endothelin-1 (ET-1) 1160/100U (R&D, Whitehead Scientific) and incubated for 45 min before treated with the four water extracts (50 μg/mL) and positive control (25 μg/mL) ambrisentan (SML2104, Sigma-aldrich), an endothelin-1A receptor inhibitor over 48 h in media supplemented with 1% FBS and 1% penicillin–streptomycin. This medium was used to prepare and dilute all the treatment and drugs. Control cells were induced with ET-1 but not treated, while non-induced control cells (No-ET) were neither induced with ET-1 nor treated.

#### Mitochondrial activity

To test for mitochondrial activity, 1 × 10^4^ cells were seeded in 96 well plates (NEST, Whitehead scientific), deprived of serum the same way as above with serum-free DMEM over 18 h prior to inducing the cells with ET-1 100 nM for 45 min. Then cells were treated with the four extracts and positive controls over 48 h in the presence of 1% FBS DMEM as above. Control cells were ET-1-induced but not treated and NO-ET1 cells were neither induced with ET-1 nor treated. Mitochondrial activity was measured using the Resazurin assay kit AR002 (R & D, Whitehead scientific) according to the manufacture manual. Viability of cells in percentages was calculated using the formula: % Viability = ((Sample Absorbance/control Absorbance) × 100). The experiments were performed in triplicate and repeated in three different times.

#### Cell surface area measurements

After 48 h treatment, the cells on cover slips were stained with haematoxylene and eosin staining and mounted on the glass slide. Images of morphological changes were taken with a light microscopy Olympus BX63 using 20 µm lenses and the cells were analysed for cell size width measurements using CellSens Dimension 1,12 software. The surface area of cells from each group (60–80 cells/group) were determined and compared with the ET-1 control cells. The results showed represented analysis from three independent experiments.

#### BNP concentration measurements

The effects of the extracts on brain natriuretic peptide (BNP) concentrations were determined and quantified using the Brain Natriuretic Peptide (BNP) Enzyme Immunoassay (EIA) (RAB0386, Sigma-aldrich) following the manufacture instructor manual on the cell culture medium after 48 h treatment. Concentration of BNP levels in the samples were calculated from a standard curve.

#### TNF-α concentration measurement

The effects of the extracts on TNF-α levels after 48 h treatment were determined on the culture media using the rat tumor necrosis factor alpha (TNF-α) ELISA kit (E-EL-R0019, Elabscience, Biocom Africa) according to the manufacture manual protocol. Concentrations of rat TNF-α concentration in the cell culture media samples were calculated from the standard curve.

#### Intracellular ROS measurements

To measure the reactive oxygen species (ROS) generated by the cells induced with ET-1 and treated with the four extracts, the cells were seeded in 96 wells and deprived with serum for 18 h. Thereafter the cells were induced with ET-1 over 2 h before treated with 50 µg/mL concentrations of the four water mushroom extracts and 25 µg/mL ambrisentan for 1 h. Fluorometric Intracellular ROS assay kit (Green Fluorescence) MAK 143 (Sigma-Adrich) was used according to manual instructions to detect intracellular ROS (especially superoxide and hydroxyl radicals) in live cells with a green fluorescence intensity at λex = 485/20520/25 nm on ET-1-induced cardiomyocytes. Fluorescence pictures of intracellular ROS production were obtained with using a 10 µm lenses. The experiment were repeated in three different times.

#### Determining the rate of cell growth

In order to determine the effects of extracts on rate of cell growth, the absorbance in each well was measured before the cells were stimulation ET-1 and also after 2 h ET-1-stimulation and 1 h treatment with the extracts (50 μg/mL) and positive control (25 μg/mL) ambrisentan. The difference in growth rate was determined by subtracting the absorbance measured before exposure from absorbance after exposure and treatment. The percentage of rate of cell survival and growth for each sample was calculated using the formula: % Viability = ((Sample difference absorbance/sample-control) × 100) where sample difference absorbance = (absorbance of sample before exposure − absorbance after exposure and treatment); and sample-control = absorbance of the sample before exposure. The effects of the extracts on rate of cell growth was determined for each samples and compared with the control ET-1 treatment and No-ET1 samples. The effects of the extracts on the rate of growth after 1 h treatment were also determined after 12, 24 and 48 h. The experiments were performed in three different conditions.

#### Protective effects of extracts on TNF-α-induced apoptosis

The effect of the four water extracts on the TNF-α-induced cardiomyocyte injury and cell death was determined by using method of^33^ with modifications. Briefly, 1 × 10^5^ cells were seeded into 96 well plates and exposed for 18 h to sera free medium as above after 24 h. The cardiomyocytes were induced with rat TNF-α (250 pg/mL) from the (TNF-α) ELISA kit (E-EL-R0019, Elabscience, Biocom Africa) for 2 h before treatment with extracts (25 and 50 µg/mL) and positive control quercetin (12.5 and 25 µg/mL) and incubated over 24 h. After 24 h mitochondrial activity was measured using Resazurin assay kit (AR002, R & D, Whitehead Scientific) according to the manufacture manual and viability of cells in percentages was calculated same as in “Mitochondrial activity” section. The experiments were performed in triplicate and repeated in three different times.

### Statistical analysis

Statistically significant values were compared using one way ANOVA analysis of variance using an interactive statistical program (Sigmastat, SPSS version 26, USA). Normality test was performed using Shapiro–Wilk and equal variance test of Brown-Forsythe. Results are expressed as mean ± standard deviations and the *p* value of ≤ 0.050 was considered statistically significant. The hot-water and cold-water extracts of *P. cubensis* are referred to as GH and GC respectively while the hot-water and cold-water of *Pan cyanescence* are referred to as PH and PC in the study.

## References

[1] Tsutsui H, Tsuchihashi-Makaya M, Kinugawa S, Goto D, Takeshita A (2006). Clinical characteristics and outcome of hospitalized patients with heart failure in Japan. Circ. J..

[2] McMurray JJV, Stewart S (2002). The burden of heart failure. Eur. Heart J..

[3] Mbakwem A, Aina F, Amadi C (2016). Depression in patients with heart failure: Is enough being done?. Cardiac. Fail. Rev..

[4] Guzman G, Allen JW, Gartz J (1998). A worldwide geographical distribution of the neurotropica fungi, an analysis and discussion. Ann. Mus. Civ. Rovereto.

[5] Carhart-Harris RL (2016). Psilocybin with psychological support for treatment-resistant depression: an open-label feasibility study. Lancet Psychiatry.

[6] Kraehenmann R (2015). Psilocybin-induced decrease in amygdala reactivity correlates with enhanced positive mood in healthy volunteers. Biol. Psychiatry.

[7] Hasler F, Grimberg U, Benz MA, Huber T, Vollenweider FX (2004). Acute psychological and physiological effects of psilocybin in healthy humans: a double-blind, placebo-controlled dose-effect study. Psychopharmacology.

[8] Amsterdam JGC, Opperhuizen A, van den Brink W (2011). Harm potential of magic mushroom: a review. Regul. Toxicol. Pharmacol..

[9] Archer CR, Robinson EL, Drawnel FM, Roderick HL (2017). Endothelin-1 promotes hypertrophic remodelling of cardiac myocytes by activating sustained signalling and transcription downstream of endothelin type A receptors. Cell. Signal..

[10] Ahuja P, Sdek P, MacLellan WR (2007). Cardiac myocyte cell cycle control in development, disease, and regeneration. Physiol. Rev..

[11] Dorn GW, Robbins J, Sugden PH (2003). Phenotyping hypertrophy: eschew obfuscation. Circ. Res..

[12] Clerk A (2007). Signaling pathways mediating cardiac myocyte gene expression in physiological and stress responses. J. Cell. Physiol..

[13] Yorikane R, Sakai S, Miyauchi T, Sakurai TY, Goto K (1993). Increased production of endothelin-1 in the hypertrophied rat heart due to pressure overload. FEBS Lett..

[14] Higazi DR (2009). Endothelin-1-stimulated InsP_3_-induced Ca^2+^ release is a nexus for hypertrophic signaling in cardiac myocytes. Mol. Cell.

[15] Singh P (2015). Sulforaphane protects the heart from doxorubicin-induced toxicity. Free Radic. Biol. Med..

[16] Levine B, Kalman J, Mayer L, Fillit HM, Packer M (1990). Elevated circulating levels of tumor necrosis factor in severe chronic heart failure. N. Engl. J. Med..

[17] Chen YF (2017). Tanshinone-induced ERs suppresses IGFII activation to alleviate Ang-II mediated cardiac hypertrophy. J. Recept. Signal Transduct..

[18] Barta T (2018). Endothelin-1-induced hypertrophic alterations and heme oxygenase-1 expression in cardiomyoblasts are counteracted by beta estradiol: in vitro and in vivo studies. Naunyn-Schmiedeberg’s Arch. Pharmacol..

[19] Schumacher SM, Naga Prasad SV (2018). Tumor necrosis factor-α in heart failure: an updated review. Curr. Cardiol. Rep..

[20] Pant N, Paudel KR, Parajuli K (2016). Reactive oxygen species: a key hallmark of cardiovascular disease. Adv. Med..

[21] Zhou L, Zhou LT, Pannell BK, Ziegler A, Best TM (2015). Biological and physiological role of reactive oxygen species-the good, the bad and the ugly. Acta Physiol..

[22] Bustillos RG (2014). Mycochemical profile of mycelia and fruiting body of *Panaeolus cyanescens* and its optimal submerged culture conditions for antioxidant properties. Int. J. Pure Appl. Biosci..

[23] Michae L (2000). Cell cycle delay and apoptosis are induced by high salt and urea in renal medullary cells. Am. J. Physiol. Renal Physiol..

[24] Teringova E, Tousek PJ (2017). Apoptosis in ischemic heart disease. J. Transl. Med..

[25] Krown KA (1996). Tumor necrosis factor-α-induced apoptosis in cardiac myocytes. Involvement of the sphingolipid signalling cascade in cardiac cell death. J. Clin. Investig..

[26] Shin S-Y (2014). The switching role of B-adrenergic receptor signaling in cell survival or death decision of cardiomyocytes. Nat. Commun..

[27] Gordon JW, Shaw JA, Kirshenbaum LA (2011). Multiple facets of NF-κB in the heart: to be or not to NF-κB. Circ. Res..

[28] Abbate A (2006). Acute myocardial infarction and heart failure: role of apoptosis. Int. J. Biochem. Cell Biol..

[29] Wu X-Y, Luo A-Y, Zhou Y-R, Ren J-H (2014). N-acetylcysteine reduces oxidative stress, nuclear factor-κB activity and cardiomyocyte apoptosis in heart failure. Mol. Med. Rep..

[30] Dhanasekaran D (2020). Taxanomic identification and bioactive compounds characterization of *Psilocybe cubensis* DPT1 to probe its antibacterial and mosquito larvicidal competency. Microb. Pathog..

[31] Nkadimeng SM, Nabatanzi A, Steinmann CML, Eloff JN (2020). Phytochemical, cytotoxicity, anti-inflammatory effects of *Psilocybe natalensis* magic mushroom. Plants.

[32] Goncalves GK (2018). Neonatal cardiomyocyte hypertrophy induced by endothelin-1 is blocked by estradiol acting on GPER. Am. J. Physiol. Cell Physiol..

[33] Zhao M (2015). Acetylcholine attenuated TNF-α-induced apoptosis in H9C2 cells: role of calpain and the p38-MAPK pathway. Cell. Physiol. Biochem..

